# Vectors Encoding Seven Oikosin Signal Peptides Transfected into CHO Cells Differ Greatly in Mediating *Gaussia* luciferase and Human Endostatin Production although mRNA Levels are Largely Unaffected

**Published:** 2007-12-11

**Authors:** Christiane Tröße, Hanne Ravneberg, Beate Stern, Ian F. Pryme

**Affiliations:** 1 UniTargetingResearch AS, Thormøhlensgt. 51, N-5006 Bergen, Norway; 2 Department of Biomedicine, University of Bergen, Jonas Lies vei 91, N-5009 Bergen, Norway

**Keywords:** Gaussia princeps, Oikopleura dioica, signal peptide, 3′UTR, oikosin, endostatin

## Abstract

The signal peptide of the luciferase secreted by the marine copepod *Gaussia princeps* has been shown to promote high-level protein synthesis/secretion of recombinant proteins, being far superior to mammalian counterparts. The main aim of the present study was to investigate the effects of seven selected signal peptides derived from oikosins, house proteins of the marine organism *Oikopleura dioica,* on synthesis/secretion of recombinant proteins. Vector constructs were made in which the coding regions of two naturally secreted proteins, *Gaussia* luciferase and human endostatin (hEndostatin), were “seamlessly” fused to the signal peptide coding sequences of interest. CHO cells were transfected with the plasmids and populations of stably transfected cells established. The amounts of reporter proteins in cell extract and medium samples were determined and the results compared to those obtained from cells stably transfected with a reference vector construct. In addition, the amounts of luciferase or hEndostatin encoding mRNAs in the cells were determined and related to the protein levels obtained. The levels of reporter protein produced varied greatly among the seven oikosin signal peptides tested. Whereas the oikosin 1 signal peptide resulted in about 40% production of *Gaussia* luciferase compared to the reference construct, oikosins 2–7 were extremely ineffective (<1%). mRNA levels were not dramatically affected such that inadequate availability of transcript for translation was not the underlying reason for the observations. The oikosin 1 signal peptide was also the most effective regarding synthesis/secretion of hEndostatin. No secreted product was observed using the oikosin 3 signal peptide. Interestingly, the molecular weight of hEndostatin in cell extracts prepared from cells transfected with oikosin 2 and 3 constructs was higher than that using the oikosin 1 signal peptide. The overall findings indicate that the signal peptide affects the efficiency of protein synthesis and secretion through a mechanism operating at the post-transcriptional level. The results described here provide substantial support to our previous observations which suggested that the choice of the signal peptide is imperative when aiming to achieve optimal synthesis and secretion of a recombinant protein using transfected mammalian cells.

## Introduction

The biotechnological industry today faces a rapidly growing demand for recombinant proteins. Most of them are to be used as pharmaceuticals, e.g. human growth factors, antibodies, hormones, blood coagulation factors and cytokines. Since they are for human use there is a requirement for high quality with regard to their purity and functionality. It is extremely important that recombinant proteins have no immunogenic effect in humans, which is mainly affected by their glycosylation pattern.

Several protein expression systems have been established to meet these demands. The most important ones for the pharmaceutical industry have been reviewed by Schmidt (2004). The choice of the proper expression system depends on the attributes of the protein to be expressed and should be carefully evaluated. Generally, the ability to generate proteins with proper folding and glycosylation patterns increases with higher phylogenetic relationship between the host and the origin of the protein to be expressed.

Bacterial systems, with the gram-negative *E. coli* as the most popular host, are well characterised, relatively cheap to grow, easy to handle and provide high production rates. However, they have the disadvantages that proper folding of heterologous mammalian proteins is not guaranteed and that the potential and capacity for posttranslational modifications is limited or non-existent, as is the case for glycosylation. Moreover, recombinant proteins are mostly produced as inclusion bodies, which makes tedious cell rupture, denaturation and refolding procedures necessary in order to purify the intact product.

More similar to human cells are eukaryotic expression systems. Yeasts offer growth and production rates similar to bacterial systems and possess in addition complex posttranslational modification pathways. But as hyperglycosylation can occur, the biological activity of the expressed protein can be affected.

Insect cells transfected with baculovirus produce large amounts of protein and are easier to operate than mammalian cell cultures. However, incomplete posttranslational modification and difficult up-scaling properties of insect cells means that industry favours the use of mammalian systems. Approved cell lines for the production of pharmaceutical proteins are for example Chinese hamster ovary (CHO) and Baby hamster kidney (BHK) cells. Culture of mammalian cell lines is less efficient and more cost-intensive than the previously mentioned host systems, but this is compensated by the quality of the product. They provide the highest capacities for and the most complete spectrum of posttranslational modification.

Over the past decades, the productivity of such mammalian cell culture expression systems has been highly increased. This was mainly achieved by improving growth conditions for the cells (better medium composition and process control), but also by genetic engineering of the protein-encoding vector, (e.g. stronger promoter/enhancer sequences, insertion of splice sites and incorporation of specific elements that either overcome the negative position effect under integration into the host genome or amplify the copy number of the gene) (Wurm, 2004).

A recent approach in our laboratory has been to explore the potential of signal peptides as modulators of protein synthesis and secretion. In a series of studies we have tested vectors encoding a variety of signal peptides for their efficiency with respect to synthesis and secretion of reporter proteins from transfected CHO cells (Knappskog et al. 2007; Stern et al. 2007a,b). It was found that the levels varied significantly. For example, the signal peptide of the constitutively secreted serum protein albumin was expected to be very efficient since liver cells daily secrete large amounts of albumin. Surprisingly the level of synthesis/secretion of the reporter protein was strikingly low (<5%) when compared to the results obtained using a signal peptide derived from a marine organism (luciferase from the copepod *Gaussia princeps*) (Knappskog et al. 2007). The interleukin-2 (Il-2) signal peptide was also tested since this is frequently used in protein production at a commercial level and in research in gene therapy (Bamford et al. 1998; Komada et al. 1999; La Flamme et al. 1995; Liu et al. 1997; Sasada et al. 1987, 1988; Suzuki et al. 2001). Similar to the observations with albumin this also turned out to be considerably inferior to the signal peptide derived from *Gaussia* luciferase (Knappskog et al. 2007). Interestingly Zhang et al. (2005) have recently shown that modification of the basicity and hydrophobicity of the Il-2 signal peptide results in augmented secretion (2.5–3.5 fold) of placental alkaline phosphatase and human endostatin (hEndostatin) from transfected MDA-MB-435 cells.

The observation that the human albumin signal peptide functioned poorly in CHO cells was surprising considering its important role in the liver where large amounts of albumin are synthesised and secreted into the blood every day. In an experiment using a hepatic cell line (HepG2) where constructs containing the coding sequences for *Gaussia* luciferase or albumin signal peptides were fused to the coding region of the luciferase, and constructs stably transfected into cells, and both medium samples and cell extracts assayed for *Gaussia* luciferase activity, then almost identical results were obtained as seen for CHO cells (Knappskog et al. 2007). It was therefore apparent that the albumin signal peptide did not operate more effectively in a cell line of hepatic origin than in CHO cells. Interestingly, the *Gaussia* luciferase signal peptide of marine origin functioned very successfully in a cell line of human derivation.

In a recent experiment (results not shown) a second reporter protein was tested (luciferase from *Vargula hilgendorfii*) where the coding regions of either the homologous signal peptide or that derived from *Gaussia* luciferase were fused to the coding region of the luciferase. The construct otherwise contained the 5′ and 3′UTRs derived from the *Vargula* luciferase gene. Assays for *Vargula* luciferase activity in cell extracts and medium samples from stably transfected cell populations showed that the construct containing the *Gaussia* luciferase signal peptide coding region was 30%–50% more effective in synthesis/secretion of *Vargula* luciferase than the native sequence. It is thus evident that the introduction of a “foreign” sequence of nucleotides coding for a heterologous signal peptide can actually have a positive effect in terms of the amount of recombinant protein produced.

Signal peptides derived from luciferases of two marine organisms, *Vargula hilgendorfii* and *Metridia longa*, proved to be less than 40% as effective compared to the *Gaussia* luciferase signal peptide in generating *Gaussia* luciferase in transfected CHO cells (Stern et al. 2007b). In similar studies using the signal peptides originating from human trypsinogen 2 and chymotrypsinogen, these were seen to be about 60%–70% as effective as the *Gaussia* luciferase signal peptide (Stern et al. 2007b). The conclusion from the above observations is that a signal peptide derived from a marine organism can operate more effectively than those of mammalian origin when tested for the efficient production of a recombinant protein using a mammalian cell line. Two prokaryotic signal peptides were also chosen in order to compare their effectiveness with those of marine/mammalian origin (Stern et al. 2007b). The signal peptide of killer toxin K28, a preprotoxin from the yeast virus M28, allows the efficient secretion of protein EGFP from different yeast strains (Eiden-Plach et al. 2004), making it a suitable candidate for testing in mammalian cells. The signal peptide of Slmj 1 from *Methanococcus jannaschii*. an extreme thermophile methanogenic Archaebacterium, living at deep sea levels under high-pressure conditions, was also considered to be an interesting contender (Akca et al. 2002). Both signal peptides, however, proved to be extremely ineffective, producing 0.01% and 10%, respectively, of the levels of *Gaussia* luciferase produced in CHO cells transfected with the construct containing the coding region of the *Gaussia* luciferase signal peptide (Stern et al. 2007b). There is, therefore, considerable variation between individual signal peptides with their respective abilities to support synthesis/secretion of recombinant proteins.

Although powerful tools exist for the identification of secretion-provoking signal peptides of proteins, like the SignalP server (Nielsen et al. 1997; Bendtsen et al. 2004; Emanuelsson et al. 2007) or the PSORT program family (Nakai and Horton, 1999; www.psort.org), these devices do not make any prognosis about the effectiveness of a particular signal peptide. It is thus evident that little is still known about the features that govern the performance of a given signal peptide. This is also illustrated by recent observations that have shown that single amino acid mutations in mammalian signal peptides can have a major effect on their functionality (Zhang et al. 2005).

Acting on the assumption that high-level secretion of a certain protein, or protein secretion under difficult environmental conditions is mediated by an “effective” signal peptide, signal peptides derived from the marine organism *Oikopleura dioica* were chosen for this study. *Oikopleura* belongs to the urochordate Appendicularians, which secrete a very complex mucous extracellular structure, the house, to filter food particles from seawater. The house is replaced every 3–4 hours, being produced by a fixed number of cells forming a monolayer covering the trunk of the animal, the oikoplastic epithelium. The house is composed of at least 20 polypeptides, some of them highly glycosylated, and its different components are secreted by specialised distinctive cells of the underlying epithelium. The house proteins are called oikosins and are divided into seven families (Spada et al. 2001; Thompson et al. 2001). It is known that the cells of the oikoplastic epithelium undergo DNA amplification as the animal grows, but it is unlikely that this is the only reason for the tremendous synthetic and secretory activity of these cells. In this study the signal peptides derived from the seven oikosin proteins were tested for their effect on production of two reporter proteins, *Gaussia* luciferase and human endostatin (hEndostatin).

## Materials and Methods

### Vector design and construction

All vector constructs generated in this study were derivatives of pTRE2hyg (Clontech). A seamless cloning method (based on that described by Chen et al. 2000) was used to introduce the desired genetic elements into the vector backbone. In the abbreviated nomenclature for the constructs p is followed by a letter code system where G refers to *Gaussia princeps* luciferase (EMBL accession no. AY015993), G* to *Gaussia* luciferase with humanised codon usage (Tannous et al. 2005), E to human endostatin (EMBL accession no. AW080065), Epo to human erythropoietin (EMBL accession no. E00630) and Oik 1–7 to oikosin proteins 1–7 derived from *Oikopleura dioica* (Seo et al. 2001). Reading from left to right the first letter refers to the source of the 5′UTR (G in all constructs), then follows the code for the signal peptide coding sequence (G*, Epo or Oik 1–7), then that for the coding sequence of the reporter protein (G* or E), and finally the source of the 3′UTR (G), in all constructs. The following constructs were prepared: pGEpoG*G, pGOik1-7G*G, pGG*EG, pGOik1-3EG and pGG*G*G was used as a control. Prediction of SP cleavage was performed *in silico* using the SignalP server (Emanuelsson et al. 2007).

#### Cultivation and transfection of CHO cells

Cultures of CHO AA8 Tet-Off cells (Clontech) were grown at 37 °C in a humidified atmosphere of 5% CO_2_ in 25 or 75 cm^2^ cell culture flasks until they reached 80%–90% confluency. Transfection was performed as described by Knappskog et al. (2007).

### Harvesting samples and luciferase measurement

Samples of transiently transfected cells were taken 24 hours after transfection. Samples of stably transfected cell populations were taken after four weeks of selection with Hygromycin B (Roche). Prior to taking samples, stably transfected cell populations were plated out on 6 well plates at a density of 2 × 10^5^ cells per well. On the following day the medium was removed and 1 ml fresh medium was added. Samples were taken 30 hours after adding the medium. For further details and a description of the assay for *Gaussia* luciferase see Knappskog et al. (2007).

#### Immunoblotting for detection of hEndostatin

Electrophoresis and the procedure used for detection of hEndostatin by immunoblotting was performed as described by Knappskog et al. (2007).

## mRNA Isolation and Northern Blotting

Total RNA was prepared from populations of stably transfected CHO cells using the Trizol reagent according to the directions provided by the manufacturer (Invitrogen). 5 × 10^6^ cells per dish were plated out on 15 cm cell culture dishes in a total of 20 ml medium per dish. RNA isolation commenced approximately 48 hours after plating out cells. Probes were prepared, and Northern blotting carried out, as reported previously (Knappskog et al. 2007).

## Results

### Effects of seven oikosin signal peptides on *Gaussia* luciferase production

Nine vector constructs, each harbouring a secretion cassette containing both the *Gaussia* luciferase 5′ and 3′UTR, the humanised *Gaussia* luciferase (G*) coding sequence and the coding sequence of one of the signal peptides to be tested were examined in two independent transfection and selection experiments. Samples of both transiently and stably transfected cells were taken and analysed for luciferase activity. The results obtained from stably transfected cell lines are considered more reliable and hence only these are presented here. In general, however, the results from transient transfection mirrored those in stably transfected cells. This was as observed by Knappskog et al. (2007) where values of luciferase activity were normalised with those obtained from transfection efficiencies in experiments using a firefly-luciferase encoding plasmid.

**Figure 1:**
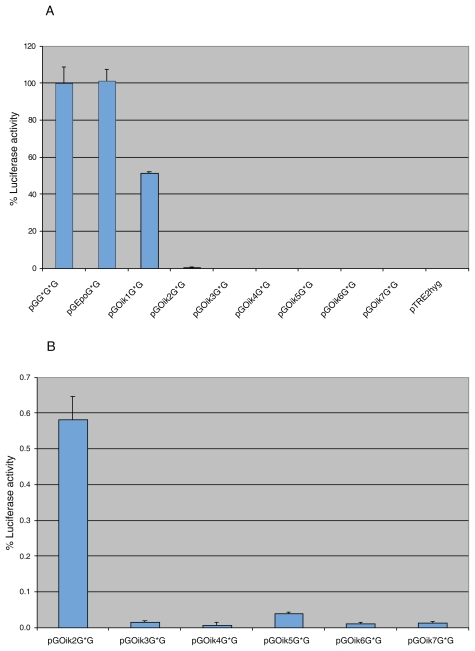
**A.** Luciferase activity in medium samples of CHO cells stably transfected with vectors encoding nine different signal peptides compared to that in medium from cells stably transfected with pGG*G*G, which was set to 100%. The results are presented as the mean of four measurements per construct from two experiments, the error bars indicating the standard deviation from the mean. The empty vector pTRE2hyg served as a negative control. **B.** Results from panel A for oikosin 2–7 signal peptides, presented on an expanded scale, where luciferase activity was <0.6% of the activity found in medium from cells transfected with pGG*G*G. For vector nomenclature see Materials and Methods.

Figure 1A shows the levels of *Gaussia* luciferase activity in medium samples from the cells transfected with the various constructs. To be able to compare results obtained from different constructs, values using pGG*G*G were set as 100% and the luciferase activity of cells transfected with the vector constructs generated in this study is presented as a percentage of this value.

The signal peptide derived from Epo was seen to be as effective as that from *Gaussia* luciferase. Of the seven oikosin signal peptides tested, six resulted in very low levels of luciferase activity (<1%) when compared to that derived from *Gaussia* luciferase (Fig. 1). Only cells transfected with pGOik1G*G showed an appreciable level of luciferase activity in the cell culture medium, amounting to 45% of that achieved using the *Gaussia* luciferase signal peptide (Fig. 1A). With respect to intracellular luciferase activity, cells transfected with constructs containing the coding region of Epo and oikosin 1 signal peptides exhibited similar levels, while oikosins 2–7 gave very low levels of activity (Table 1).

## Northern Blot Analysis of *Gaussia* luciferase mRNA Levels

**Figure 2:**
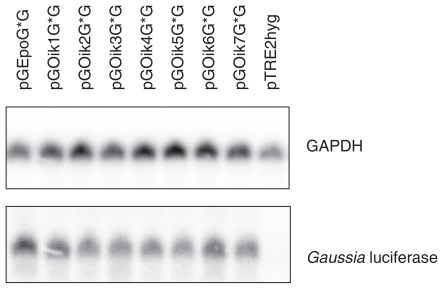
Northern blot showing GAPDH mRNA and *Gaussia* luciferase mRNA in total RNA samples from populations of stably transfected CHO cells. For vector nomenclature see Materials and Methods.

A possible explanation for the production of greatly varying amounts of reporter protein could have been major differences between mRNA levels. In order to compare the relative amounts of mRNA transcripts of the reporter gene present in the stably transfected cells, total RNA was isolated from the individual cell extracts and subjected to gel electrophoresis. The RNA was subsequently blotted onto a nitrocellulose membrane via capillary transfer, and mRNA was visualised using radioactively labelled DNA probes against GAPDH and *Gaussia* luciferase (Fig. 2). The amounts of mRNA bound by the probes were quantified by autoradiography using a Packard Instant Imager (see Table 1). GAPDH mRNA served as a reference for the quantitation of the reporter protein mRNA in a sample. When the amount of *Gaussia* luciferase mRNA produced by the reference construct, in this case Epo, was set to 100% then levels of transcript found in cells transfected with the seven oikosin signal peptides ranged between 48 and 72%. It is thus evident that cells transfected with the oikosin constructs did contain less *Gaussia* luciferase mRNA than cells transfected with the reference construct. The levels of mRNA, however, were not sufficiently low to explain the extremely limited production of *Gaussia* luciferase observed using oikosin 2–7 signal peptides (Table 1).

## hEndostatin Production using Different Signal Peptides

In order to investigate whether or not the effect of a signal peptide is protein specific, a second recombinant reporter protein, namely hEndostatin, was chosen for comparative studies with selected oikosin signal peptides.

From Figure 1 it is evident that there were major differences in *Gaussia* luciferase production depending on which of the seven oikosin signal peptides was used. The levels of luciferase activity in medium samples for oikosin 1–3 signal peptides were about 45%, 0.6% and 0.1% respectively, related to that of the *Gaussia* luciferase signal peptide construct (Fig. 1B). Since these demonstrated such diverse differences with respect to *Gaussia* luciferase synthesis/secretion they were chosen for studies on hEndostatin production.

Vector constructs containing the hEndostatin coding sequence and the coding sequence of the signal peptides from oikosin 1, 2 and 3 were generated and CHO cells were transiently transfected with the plasmids, medium samples were taken and cell extracts were prepared. The total protein concentration in the cell extract samples was determined prior to gel electrophoresis. To be able to compare the intensity of the bands directly, equal amounts of sample were loaded on the gel and the medium samples were proportionally applied.

**Figure 3:**
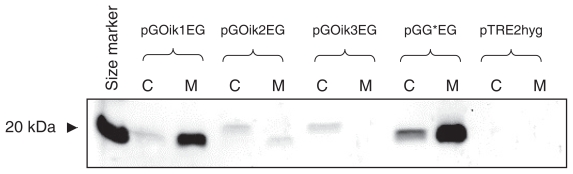
Western blot of cell extracts and medium samples of CHO cells transiently transfected with plasmids containing the coding regions of the signal peptides derived from oikosins 1–3 fused to the hEndostatin coding sequence. Samples of cells transfected with the empty vector pTRE2hyg served as a negative control. C = cell extract, M = medium sample. For vector nomenclature see Materials and Methods.

Western blot analysis revealed that the hEndostatin content was highest in medium samples and extracts of cells containing the construct that included the *Gaussia* luciferase signal peptide (see Fig. 3). In comparison the medium samples obtained from cells transfected with the oikosin 1 signal peptide construct contained about half as much hEndostatin. Transfection with pGOik2EG resulted in production of only very small amounts of hEndostatin whereas cells transfected with pGOik3EG showed no detectable amount in the medium sample (Fig. 3).

It can be seen from Figure 3 that bands of different sizes appear on the blot. Cells that contained either the *Gaussia* luciferase signal peptide or that derived from oikosin 1 showed bands in the medium and cell extracts of identical molecular weight. The bands at 20 kDa represent correctly processed hEndostatin. In contrast, a band clearly larger than 20 kDa is apparent in the extracts prepared from cells transfected with constructs containing either the oikosin 2 or oikosin 3 signal peptides. The band in the medium from oikosin 2 transfected cells was of the same size as that observed when signal peptides derived from *Gaussia* luciferase and oikosin 1 were tested. As expected, no hEndostatin was found in samples taken from cells transiently or stably transfected with the empty vector pTRE2hyg or untransfected CHO cells (not shown).

## Discussion

The main objective of this work was to compare the effects of seven oikosin signal peptides on synthesis/secretion of two recombinant proteins (*Gaussia* luciferase and hEndostatin) in transfected CHO cells with results obtained using reference signal peptides, namely those derived from codon optimised *Gaussia* luciferase and human Epo. The relation between the amount of secreted protein and the corresponding mRNA produced was also studied. All vector constructs used were identical, apart from the inserted secretion cassette. Use of seamless cloning made it possible to assemble secretion cassettes without including any linker sequences between their individual components. In order to ensure reproducible results not depending on transfection efficiency, populations of stably transfected cells were established and used for the determination of the amount of secreted protein.

As can be seen in Figure 1A and B, *Gaussia* luciferase activity was measurable in medium samples of cells stably transfected with all vector constructs containing coding regions of oikosin signal peptides. Although the Epo signal peptide proved to be as effective as that derived from *Gaussia* luciferase, there were very large variations in the levels of luciferase activity found in medium samples of cell populations stably transfected with vector constructs containing the oikosin 1–7 signal peptide coding regions. Four of the oikosin signal peptides (oikosins 3, 4, 6 and 7) resulted in amounts of secreted luciferase which were only 0.01% of that achieved using either the *Gaussia* luciferase or Epo signal peptides. Oikosin 1 signal peptide was very different to the others in the family since a level of 45% of that using the reference signal peptide was achieved. Immunoblotting had earlier shown that low levels of luciferase activity were mirrored by weak bands of luciferase protein such that the presence of large amounts of inactive product did not accompany low levels of activity (Knappskog et al. 2007). It was thus assumed that large amounts of inactive enzyme was not the explanation for the results described here.

The findings reported in this paper support those in previous studies (Knappskog et al. 2007; Stern et al. 2007a, b), where the efficiency of individual signal peptides was found to vary greatly. It was quite unexpected, though, to find that six of the seven oikosin signal sequences resulted in production of such strikingly low levels of luciferase activity when compared to the *Gaussia* luciferase signal peptide. The performance of a signal peptide is, therefore, not necessarily predictable from the features of the corresponding protein or the role this protein plays in the organism from which it is originally derived.

An important observation was that the construct containing the oikosin 1 signal peptide, in contrast to constructs containing oikosin 2–7 signal peptides, caused the secretion of large amounts of both *Gaussia* luciferase and hEndostatin. It is also interesting to note that the oikosin 1 and 3 signal peptides varied by many magnitudes in the amount of luciferase secreted (45% and 0.01%, respectively of reference values), also varied in the respective amounts of endostatin secreted.

In order to investigate whether the observed effects caused by the incorporation of coding regions for signal peptides into RNA transcripts take place at the stage of transcription or translation, the levels of *Gaussia* luciferase mRNA in stably transfected cell populations were determined. The levels of mRNA varied from 48%–64% (oikosin signal peptides 2 and 7) to 72% (oikosin 1) of that achieved with the signal peptide derived from Epo, although the vector constructs the cells had been transfected with contained identical promoter and polyadenylation sequences and differed only in the nature of the signal peptide coding sequence. Similar results were obtained by Knappskog et al. (2007) who demonstrated that when coding sequences of Il-2 and albumin signal peptides were fused to the coding region of *Gaussia* luciferase then relatively low levels of active product (37 and 2%, respectively, compared to the *Gaussia* luciferase signal peptide set as 100%) were obtained but large amounts of mRNA (up to 90%) were detected by Northern blotting. It is not yet known whether the observations can be explained by different levels of translatability of the mRNA.

Another possible explanation for the results presented here is the issue of mRNA targeting. If the mRNA is not targeted correctly to the ER, or if the targeting is not efficient/fast enough, then this may result in greatly reduced levels of protein synthesis and secretion. The results obtained with hEndostatin may suggest that mRNA targeting could be involved. In the case of oikosin 1 correct targeting of mRNA to membrane-bound polysomes has evidently occurred, the signal peptide then being cleaved off following translation and efficient secretion of hEndostatin taking place. With oikosin 2 the situation was somewhat different since hEndostatin in the cell extract had a higher molecular weight than the secreted product, perhaps indicating problems with respect to cleavage of the signal peptide. A higher molecular weight product was also identified in the cell extract when using the oikosin 3 signal peptide but here no secretion into the medium was observed. This was surprising taking into consideration the fact that the bands of hEndostatin in the cell extracts prepared from cells transfected with pGOik2EG and pGOik3EG were of similar intensity. Taken together these results would suggest that using the oikosin 1 signal peptide then both correct targeting of mRNA and efficient cleavage of the signal peptide were able to occur. This was in contrast to oikosin 3 where a non-cleaved product was apparently observed in the cell extract and no secreted product was detected, perhaps suggesting ineffective mRNA targeting. In the case of oikosin 2 an “intermediate” situation was apparent since secretion of authentic hEndostatin did occur although the cell extract appeared to contain non-cleaved product. Further work will be required to examine this in detail.

The analysis of hEndostatin by immunoblotting indicated the presence of approximately the same amount of protein—cleaved or uncleaved—in cell extracts of cells transiently transfected with constructs containing the oikosin 1, 2 and 3 signal peptide coding regions. On the other hand measurement of hEndostatin revealed high levels in the media from cells transfected with pGOik1G*G compared to those transfected with pGOik2G*G or pGOik3G*G. The reason for this is not yet understood.

To our knowledge this is the first report where a series of signal peptides derived from a family of proteins has been tested and compared for their relative abilities to support the synthesis and secretion of recombinant proteins. The results described here clearly illustrate that the actual choice of signal peptide is imperative when designing a secretion vector for optimal production of recombinant proteins, and furthermore, they stress the fact that it is possible that there is important information within the coding sequence of the signal peptide, or the signal peptide itself. A control mechanism at the post-transcriptional level appears to be implicated.

## Figures and Tables

**Table 1:** Characteristics of the cell populations stably transfected with the indicated vectors. For vector nomenclature see Materials and Methods. Levels of luciferase activity both in medium and cell extract samples are given in relation to the luciferase activity in the medium sample of cells transfected with pGEpoG*G, which is set to 100%. Luciferase mRNA has been corrected for the corresponding level of GAPDH mRNA (from Fig. 2) in the sample and values are presented in relation to those achieved with pGEpoG*G (set to 100%).

Vector	Luciferase activity in medium (%)	Luciferase activity in cell extract (%)	Luciferase mRNA (%)
pGEpoG*G	100	6.69	100
pGOik1G*G	44.6	6.67	72
pGOik2G*G	0.6	0.06	48
pGOik3G*G	0.01	0.13	56
pGOik4G*G	0.01	0.06	52
pGOik5G*G	0.04	0.04	56
pGOik6G*G	0.01	0.04	64
pGOik7G*G	0.01	0.04	48
